# Supplementary material for: Predictors of alcohol use disorder risk in young adults: Direct and indirect psychological paths through binge drinking
Source: PLoS One. 2025 May 9;20(5):e0321974. doi: 10.1371/journal.pone.0321974 (PMC12064033; doi:10.1371/journal.pone.0321974)
Supplement: S1 File — (DOCX) [file pone.0321974.s001.docx]

**Supporting Information**

**For the manuscript**

“Predictors of Alcohol Use Disorder Risk in Young Adults:
Direct and Indirect Psychological Paths through Binge Drinking”

**Table of contents**

[1. Alcohol consumption variables 2](#_Toc127451932)

[AUDIT 2](#_Toc127451933)

[Binge drinking score 4](#_Toc127451934)

[2. Intraindividual factors 5](#_Toc127451935)

[o Personality traits 5](#_Toc127451936)

[STAIT-Y anxiety scales 5](#_Toc127451937)

[BDI 6](#_Toc127451938)

[ESUL 9](#_Toc127451939)

[UPPS 10](#_Toc127451940)

[o Metacognitions 11](#_Toc127451941)

[PAMS 11](#_Toc127451942)

[NAMS 12](#_Toc127451943)

[3. Interindividual factors 13](#_Toc127451944)

[DMQ-R Short Form 13](#_Toc127451945)

[Subjective norm 14](#_Toc127451946)

[Drinking identity 14](#_Toc127451947)

# Alcohol consumption variables

| AUDIT | | | | |
| --- | --- | --- | --- | --- |
|  | Version française | Gache P, Michaud P, Landry U, Accietto C, Arfaoui S, Wenger O, Daeppen J. (2005) The Alcohol Use Disorders Identification Test (AUDIT) as a screening tool for excessive drinking in primary care: reliability and validity of a French version. *Alcoholism: Clinical and Experimental Research* **29**: 2001-2007. doi: [10.1097/01.alc.0000187034.58955.64](https://doi.org/10.1097/01.alc.0000187034.58955.64) | | |
|  | English version | Saunders JB, Aasland OG, Babor TF, DeLaFuente JR, Grant M (1993) Development of the Alcohol Use Disorders Identification Test (AUDIT): WHO collaborative project on early detection of persons with harmful alcohol consumption II. Addiction 88:791–804. | | |
| **Alcohol intake** | | | | |
| 1 | A quelle fréquence vous arrive-t-il de consommer des boissons contenant de l’alcool ? | Jamais  Une fois par mois ou moins  2-4 fois par mois  2-3 fois par semaine  4 fois ou plus par semaine | How often do you have a drink containing alcohol? | Never  Monthly or less  Two to four times a month  Two to three times a week  Four or more  times a week |
| 2 | Combien de verres (standards) contenant de l’alcool buvez-vous au cours d’une journée ordinaire où vous consommez de l’alcool ? | Un ou deux  3 ou 4  5 ou 6  7 ou 9  10 ou plus | How many drinks containing alcohol do you have on a typical day when you are drinking? | 1 or 2  3 or 4  5 or 6  7 to 9  10 or more |
| 3 | Au cours d’une même occasion, à quelle fréquence vous arrive-t-il de boire 6 verres standards ou plus? | Jamais  Moins d’une fois par mois  Une fois par mois  Une fois par semaine  Chaque jour ou presque | How often do you have six or more drinks on one occasion? | Never  Less than monthly  Monthly  Weekly  Daily or almost daily |

| **Alcohol dependence symptoms** | | | | |
| --- | --- | --- | --- | --- |
| 4 | Au cours de l’année écoulée, à quelle fréquence avez-vous constaté que vous n’étiez plus capable de vous arrêter de boire après avoir commencé ? | Jamais  Moins d’une fois par mois  Une fois par mois  Une fois par semaine  Chaque jour ou presque | How often during the last year have you found that you were not able to stop drinking once you had started? | Never  Less than monthly  Monthly  Weekly  Daily or almost daily |
| 5 | Dans les douze derniers mois, à quelle fréquence le fait d’avoir bu de l’alcool vous a-t-il empêché de faire ce que l’on attendait normalement de vous ? | Jamais  Moins d’une fois par mois  Une fois par mois  Une fois par semaine  Chaque jour ou presque | How often during the last year have you failed to do what was normally expected from you because of drinking? | Never  Less than monthly  Monthly  Weekly  Daily or almost daily |
| 6 | Dans les douze derniers mois, à quelle fréquence après une période de forte consommation, avez-vous dû boire de l’alcool dès le matin pour vous remettre en forme ? | Jamais  Moins d’une fois par mois  Une fois par mois  Une fois par semaine  Chaque jour ou presque | How often during the last year have you needed a first drink in the moming to get yourself going after a heavy drinking session? | Never  Less than monthly  Monthly  Weekly  Daily or almost daily |
| **Alcohol-related problems** | | | | |
| 7 | Dans les douze derniers mois, à quelle fréquence avez-vous eu un sentiment de culpabilité ou de regret après avoir bu ? | Jamais  Moins d’une fois par mois  Une fois par mois  Une fois par semaine  Chaque jour ou presque | How often during the last year have you had a feeling of guilt or remorse after drinking? | Never  Less than monthly  Monthly  Weekly  Daily or almost daily |

| 8 | Dans les douze derniers mois, à quelle fréquence avez-vous été incapable de vous souvenir de ce qui s’était passé la nuit précédente parce que vous aviez bu ? | Jamais  Moins d’une fois par mois  Une fois par mois  Une fois par semaine  Chaque jour ou presque | How often during the last year have you been unable to remember what happened the night before because you had been drinking? | Never  Less than monthly  Monthly  Weekly  Daily or almost daily |
| --- | --- | --- | --- | --- |
| 9 | Vous êtes-vous blessé ou avez-vous blessé quelqu’un d’autre parce que vous aviez bu ? | Non  Oui, pas dans les 12 derniers mois  Oui au cours des 12 derniers mois | Have you or someone else been injured as a result of your drinking? | No  Yes, but not in the last year  Yes, during the last year |
| 10 | Est-ce qu’un parent, un ami, un médecin ou un autre professionnel s’est déjà préoccupé de votre consommation d’alcool et vous a conseillé de la diminuer ? | Non  Oui, pas dans les 12 derniers mois  Oui au cours des 12 derniers mois | Has a relative or friend, or a doctor or other health worker been concerned about your drinking or suggested you cut down? | No  Yes, but not in the last year  Yes, during the last year |
|  | | | | |
| Binge drinking score | | | | |
|  | English version | Townshend J, Duka T. (2002) Patterns of alcohol drinking in a population of young social drinkers: a comparison of questionnaire and diary measures. *Alcohol & Alcoholism* **37**: 187-192. doi: [10.1093/alcalc/37.2.187](https://doi.org/10.1093/alcalc/37.2.187) | | |
| 1 | Lorsque vous buvez, à quelle vitesse le faîtes-vous en nombre de boissons alcoolisées par heure ? | 1/3 de verre  1/2 verre  1verre  2 verres  3 verres  4 verres  5 verres  6 verres  7 verres et + | When you drink, how fast do you drink per hour? (Here, a drink is a glass of wine, a pint of beer or a shot of spirits, straight or mixed) |  |
| 2 | Combien de fois avez-vous été saoul(e) ces 6 derniers mois ? *Être saoul(e) implique une perte de coordination, des nausées et/ou incapacité à parler clairement*… | De 0 à 200 | How many times have you been drunk in the last 6 months? By « drunk » we mean loss of co-ordination, nausea, and/or inability to speak clearly. |  |
| 3 | Quel est le pourcentage de fois où vous êtes saoul(e) lorsque vous buvez ? | De 0 à 100% | What percentage of the times that you drink do you get drunk? |  |

# Intraindividual factors

# Personality traits

| STAIT-Y anxiety scales | | | | |
| --- | --- | --- | --- | --- |
|  | Source | Spielberger C, Gorsuch R, Lushene R, Vagg P, Jacobs G. (1983) *Manual for the state-trait anxiety inventory*. Palo Alto, CA: Consulting Psychologists Press | | |
|  | Adaptation française | Bergeron, J., Landry, M., & Bélanger, D. (1976). The development and validation of a French form of the State-Trait Anxiety Inventory. *Cross-cultural anxiety*, *1*, 41-50. | | |
|  | English version | Bergua, V., Meillon, C., Potvin, O., Ritchie, K., Tzourio, C., Bouisson, J., ... & Amieva, H. (2016). Short STAI-Y anxiety scales: validation and normative data for elderly subjects. *Aging & mental health*, *20*(9), 987-995 | | |
|  | Un certain nombre de phrases que l’on utilise pour se décrire sont données ci-dessous. Lisez chaque phrase, puis cochez, parmi les modalités de réponse, celle qui correspond le mieux à ce que vous ressentez GENERALEMENT. |  |  |  |
| 1 | Je me sens de bonne humeur, aimable | 1 : Non  2 : Plutôt non  3 : Plutôt oui  4 : Oui | I feel pleasant | 1 : No  2 : Rather not  3 : Rather yes  4 : Yes |
| 2 | Je me sens nerveux(se) et agité(e). |  | I feel nervous and restless |  |
| 3 | Je me sens content(e) de moi. |  | I feel satisfied with myself |  |
| 4 | Je voudrais être aussi heureux(se) que les autres. |  | I wish I could be as happy as others seem to be |  |
| 5 | J’ai un sentiment d’échec. |  | I feel like a failure |  |
| 6 | Je me sens reposé(e). |  | I feel rested |  |
| 7 | J’ai tout mon sang froid. |  | I am calm, cool and collected |  |
| 8 | J’ai l’impression que les choses s’accumulent à un tel point que je ne peux plus les surmonter |  | I feel that difficulties are pilling up so that I cannot overcome them |  |
| 9 | Je m’inquiète à propos de choses sans importance. |  | I worry too much over something that really doesn’t matter |  |
| 10 | Je suis heureux(se). |  | I am happy |  |
| 11 | J’ai des pensées qui me perturbent. |  | I have disturbing thoughts |  |
| 12 | Je manque de confiance en moi. |  | I lack self-confidence |  |
| 13 | Je me sens sans inquiétude, en sécurité, en sûreté. |  | I feel secure |  |
| 14 | Je prends facilement des décisions. |  | I make decision easily |  |
| 15 | Je me sens incompétent(e), pas à la hauteur. |  | I feel inadequate |  |
| 16 | Je suis satisfait(e). |  | I am content |  |
| 17 | Des idées sans importance trottant dans ma tête me dérangent. |  | Some unimportant thought runs through my mind and bothers me |  |
| 18 | Je prends les déceptions tellement à cœur que je les oublie difficilement. |  | I take disappointments so keenly that I can’t put them out of my mind |  |
| 19 | Je suis une personne posé(e), solide, stable. |  | I am a steady person |  |
| 20 | Je deviens tendu(e) et agité(e) quand je réfléchis à mes soucis |  | I become tense and upset when I think about my present concerns |  |
|  | | | | |
| BDI | | | | |
|  | English version | Beck, A. T., Ward, C. H., Mendelson, M., Mock, J., & Erbaugh, J. (1961). An inventory for measuring depression. *Archives of general psychiatry*, *4*(6), 561-571. | | |
| **A** | **Humeur** |  | **Mood** |  |
| 1 | Je ne me sens pas triste. | Valeur 0 | I do not feel sad | Value 0 |
| 2 | Je me sens cafardeux ou triste. | Valeur 1 | I feel blue or sad | Value 1 |
| 3 | Je me sens tout le temps cafardeux ou triste, et je n’arrive pas en sortir. | Valeur 2 | I am blue or sad all the time and I can't snap out of it | Value 2 |
| 4 | Je suis si triste et si malheureux, que je ne peux pas le supporter. | Valeur 3 | I am so sad or unhappy that I can't stand it | Value 3 |
| **B** | **Pessimisme** |  | **Pessimism** |  |
| 1 | Je ne suis pas particulièrement découragé, ni pessimiste au sujet de l’avenir. | Valeur 0 | I am not particularly pessimistic or discouraged about the future | Value 0 |
| 2 | J’ai un sentiment de découragement au sujet de l’avenir. | Valeur 1 | I feel discouraged about the future | Value 1 |
| 3 | Pour mon avenir, je n’ai aucun motif d’espérer. | Valeur 2 | I feel I have nothing to look forward to | Value 2 |
| 4 | Je sens qu’il n’y a aucun espoir pour mon avenir, et que la situation ne peut s’améliorer. | Valeur 3 | I feel that the future is hopeless and that things cannot improve | Value 3 |
| **C** | **Sentiment d’échec** |  | **Sense of Failure** |  |
| 1 | Je n’ai aucun sentiment d’échec de ma vie. | Valeur 0 | I do not feel like a failure | Value 0 |
| 2 | J’ai l’impression que j’ai échoué dans ma vie plus que la plupart des gens. | Valeur 1 | I feel I have failed more than the average person | Value 1 |
| 3 | Quand je regarde ma vie passée, tout ce que j’y découvre n’est qu’échecs. | Valeur 2 | As I look back on my life all I can see is a lot of failures | Value 2 |
| 4 | J’ai un sentiment d’échec complet dans toute ma vie personnelle (dans mes relations avec mes parents, mon mari, ma femme mes enfants) | Valeur 3 | I feel I am a complete failure as a person (parent, husband, wife) | Value 3 |
| **D** | **Manque de satisfaction** |  | **Lack of Satisfaction** |  |
| 1 | Je ne me sens pas particulièrement insatisfait. | Valeur 0 | I am not particularly dissatisfied | Value 0 |
| 2 | Je ne sais pas profiter agréablement des circonstances. | Valeur 1 | I feel bored most of the time | Value 1 |
| 3 | Je ne tire plus aucune satisfaction de quoi que ce soit. | Valeur 2 | I don't get satisfaction out of anything any more | Value 2 |
| 4 | Je suis mécontent de tout | Valeur 3 | I am dissatisfied with everything | Value 3 |
| **E** | **Sentiment de culpabilité** |  | **Guilty feeling** |  |
| 1 | Je ne me sens pas coupable. | Valeur 0 | I don't feel particularly guilty | Value 0 |
| 2 | Je me sens mauvais ou indigne une bonne partie du temps. | Valeur 1 | I feel bad or unworthy a good part of the time | Value 1 |
| 3 | Je me sens coupable. | Valeur 2 | I feel quite guilty | Value 2 |
| 4 | Je me juge très mauvais, et j’ai l’impression que je ne vaux rien. | Valeur 3 | I feel as though I am very bad or worthless | Value 3 |
| **F** | **Haine envers soi-même** |  | **Self Hate** |  |
| 1 | Je ne suis pas déçu par moi-même. | Valeur 0 | I don't feel disappointed in myself | Value 0 |
| 2 | Je suis déçu par moi-même. | Valeur 1 | I am disappointed in myself | Value 1 |
| 3 | Je me dégoûte moi-même. | Valeur 2 | I am disgusted with myself | Value 2 |
| 4 | Je me hais | Valeur 3 | I hate myself | Value 3 |
| **G** | **Souhaits auto-punitifs** |  | **Self-punitive Wishes** |  |
| 1 | Je ne pense pas à me faire du mal. | Valeur 0 | I don't have any thoughts of harming myself | Value 0 |
| 2 | Je pense que la mort me libèrerait. | Valeur 1 | I feel I would be better off dead | Value 1 |
| 3 | J’ai des plans précis pour me suicider. | Valeur 2 | I have definite plans about committing suicide | Value 2 |
| 4 | Si je le pouvais, je me tuerais | Valeur 3 | I would kill myself if I could | Value 3 |
| **H** | **Retrait social** |  | **Social Withdrawal** |  |
| 1 | Je n’ai pas perdu l’intérêt pour les autres gens. | Valeur 0 | I have not lost interest in other people | Value 0 |
| 2 | Maintenant je m’intéresse moins aux autres gens qu’autrefois. | Valeur 1 | I am less interested in other people now than I used to be | Value 1 |
| 3 | J’ai perdu tout l’intérêt que je portais aux gens et j’ai peu de sentiments pour eux. | Valeur 2 | I have lost most of my interest in other people and have little feeling for them | Value 2 |
| 4 | J’ai perdu tout l’intérêt pour les autres, et ils m’indiffèrent totalement. | Valeur 3 | I have lost all my interest in other people and don't care about them at all | Value 3 |
| **I** | **Indécision** |  | **Indecisiveness** |  |
| 1 | Je suis capable de me décider aussi facilement que de coutume. | Valeur 0 | I make decisions about as well as ever | Value 0 |
| 2 | J’essaie de ne pas avoir à prendre de décision. | Valeur 1 | I am less sure of myself now and try to put off making decisions | Value 1 |
| 3 | J’ai de grandes difficultés à prendre des décisions. | Valeur 2 | I can't make decisions any more without help | Value 2 |
| 4 | Je ne suis plus capable de prendre la moindre décision. | Valeur 3 | I can't make any decisions at all any more | Value 3 |
| **J** | **Image du corps** |  | **Body Image** |  |
| 1 | Je n’ai pas le sentiment d’être plus laid qu’avant. | Valeur 0 | I don't feel I look any worse than I used to | Value 0 |
| 2 | J’ai peur de paraître vieux ou disgracieux. | Valeur 1 | I am worried that I am looking old or unattractive | Value 1 |
| 3 | J’ai l’impression qu’il y a un changement permanent dans mon apparence physique, qui me fait paraître disgracieux. | Valeur 2 | I feel that there are permanent changes in my appearance and they make me look unattractive | Value 2 |
| 4 | J’ai l’impression d’être laid et repoussant | Valeur 3 | I feel that I am ugly or repulsive looking | Value 3 |
| **K** | **Inhibition au travail** |  | **Work Inhibition** |  |
| 1 | Je travaille aussi facilement qu’auparavant. | Valeur 0 | I can work about as well as before | Value 0 |
| 2 | Il me faut faire un effort supplémentaire pour commencer à faire quelque chose. | Valeur 1 | It takes extra effort to get started at doing something | Value 1 |
| 3 | Il faut que je fasse un très grand effort pour faire quoi que ce soit. | Valeur 2 | I have to push myself very hard to do anything | Value 2 |
| 4 | Je suis incapable de faire le moindre travail | Valeur 3 | I can't do any work at all | Value 3 |
| **L** | **Fatigabilité** |  | **Fatigability** |  |
| 1 | Je ne suis pas plus fatigué que d’habitude. | Valeur 0 | I don't get any more tired than usual | Value 0 |
| 2 | Je suis fatigué plus facilement que d’habitude. | Valeur 1 | I get tired more easily than I used to | Value 1 |
| 3 | Faire quoi que ce soit me fatigue. | Valeur 2 | I get tired from doing anything | Value 2 |
| 4 | Je suis incapable de faire le moindre travail. | Valeur 3 | I get too tired to do anything | Value 3 |
| **M** | **Perte d’appétit** |  | **Loss of Appetite** |  |
| 1 | Mon appétit est toujours aussi bon. | Valeur 0 | My appetite is no worse than usual | Value 0 |
| 2 | Mon appétit n’est pas aussi bon que d’habitude. | Valeur 1 | My appetite is not as good as it used to be | Value 1 |
| 3 | Mon appétit est beaucoup moins bon maintenant. | Valeur 2 | My appetite is much worse now | Value 2 |
| 4 | Je n’ai plus du tout d’appétit | Valeur 3 | I have no appetite at all any more | Value 3 |

| ESUL - Loneliness | | | | |
| --- | --- | --- | --- | --- |
|  | English version | Russell, D., Peplau, L. A., & Cutrona, C. E. (1980). The revised UCLA Loneliness Scale: concurrent and discriminant validity evidence. *Journal of personality and social psychology*, *39*(3), 472. | | |
|  | Version française : | De Grace, G. R., Joshi, P., & Pelletier, R. (1993). L'Échelle de solitude de l'Université Laval (ÉSUL) : validation canadienne-française du UCLA Loneliness Scale. *Canadian Journal of Behavioural Science/Revue canadienne des sciences du comportement*, *25*(1), 12. | | |
|  | Indiquez la fréquence avec laquelle chacun des énoncés décrit bien ce que vous ressentez. Encerclez un chiffre pour chaque énoncé |  | Indicate how often you feel the way described in each of the following statements. Circle one number for each. |  |
| 1 (R) | Je me sens sur la même longueur d'ondes que les gens autour de moi | 1 - Jamais  2 - Rarement  3 - Quelque fois  4 - Souvent | I feel in tune with the people around me | 1-Never  2-Rarely  3-Sometimes  4-Often |
| 2 | Je n'ai pas assez d'ami(e)s |  | I lack companionship |  |
| 3 | II n'y a personne à qui je peux avoir recours |  | There is no one I can turn to |  |
| 4 (R) | Je ne me sens pas seul(e) |  | I do not feel alone |  |
| 5 (R) | Je sens que je fais partie d'un groupe d'amis |  | I feel part of a group of friends |  |
| 6 (R) | J'ai beaucoup de choses en commun avec les gens qui m'entourent |  | I have a lot in common \vith the people around me |  |
| 7 | Je ne me sens plus proche de quiconque |  | I am no longer close to anyone |  |
| 8 | Mes intérêts et idées ne sont pas partagés par ceux qui m'entourent |  | My interests and ideas are not shared by those  around me |  |
| 9 (R) | Je suis une personne sociable |  | I am an outgoing person |  |
| 10 (R) | II y a des gens dont je me sens proche |  | There are people I feel close to |  |
| 11 | Je me sens exclu(e) |  | I feel left out |  |
| 12 | Mes relations sociales sont superficielles |  | My social relationships are superficial |  |
| 13 | Personne ne me connaît vraiment bien |  | No one really knows me well |  |
| 14 | Je me sens isolé(e) des autres |  | I feel isolated from others |  |
| 15 (R) | Je peux m'entourer d'ami(e)s quand je le veux |  | I can find companionship \vhen I want it |  |
| 16 (R) | II y a des gens qui me comprennent vraiment |  | There are people who really understand me |  |
| 17 | Je me sens malheureux(se) d’être aussi retiré(e) |  | I am unhappy being so withdrawn |  |
| 18 | Les gens sont autour de moi et non avec moi |  | People are around me but not with me |  |
| 19 (R) | II y a des gens à qui je peux parler |  | There are people I can talk to |  |
| 20 (R) | II y a des gens à qui je peux avoir recours |  | There are people I can turn to |  |
| UPPS | | | | |
|  | Versions françaises | Billieux J, Rochat L, Ceschi G, Carré A, Offerlin-Meyer I, Defeldre A, Khazaal Y, Besche-Richard C, Van der Linden M. (2012) Validation of a short French version of the UPPS-P Impulsive Behavior Scale. *Comprehensive Psychiatry* **53**: 609-615. doi: 10.1016/j.comppsych.2011.09.001  Van der Linden, M., d'Acremont, M., Zermatten, A., Jermann, F., Larøi, F., Willems, S., ... & Bechara, A. (2006). A French adaptation of the UPPS impulsive behavior scale. *European Journal of Psychological Assessment*, *22*(1), 38-42. | | |
|  | English version | Whiteside, S.P., & Lynam, D.R. (2001). The Five Factor Model and impulsivity: Using a structural model of personality to understand impulsivity. Personality and Individual Differences, 30, 669–689 | | |
|  | Source des items Urgence positive | Cyders, M. A., Smith, G. T., Spillane, N. S., Fischer, S., Annus, A. M., & Peterson, C. (2007). Integration of impulsivity and positive mood to predict risky behavior: development and validation of a measure of positive urgency. *Psychological assessment*, *19*(1), 107 | | |
|  |  | 5 factors model | a – Premeditation  b – Positive Urgency  c – Negative Urgency  d – Sensation Seeking  e - Perseverance |  |
| 1 | D’habitude je réfléchis soigneusement avant de faire quoi que ce soit. | De 1 (Tout à fait d’accord)  à  4 (Tout à fait en désaccord) | ^a^ I usually think carefully before doing anything | From 1  (I agree strongly)  to  4  (I disagree strongly) |
| 2 (R) | Quand je suis vraiment enthousiaste, j’ai tendance à ne pas penser aux conséquences de mes actions. |  | ^b^ When I am really excited, I tend not to think of the consequences of my actions |  |
| 3 (R) | J’aime parfois faire des choses qui sont un petit peu effrayantes. |  | ^d^ I sometimes like doing things that are a bit frightening. |  |
| 4 (R) | Quand je suis contrarié(e), j’agis souvent sans réfléchir |  | ^c^ When I am upset I often act without thinking |  |
| 5 | Je préfère généralement mener les choses jusqu’au bout. |  | ^e^ I generally like to see things through to the end |  |
| 6 | Ma manière de penser est d’habitude réfléchie et méticuleuse. |  | ^a^ My thinking is usually careful and purposeful |  |
| 7 (R) | Quand la discussion s'échauffe, je dis souvent des choses que je regrette ensuite |  | ^c^ In the heat of an argument, I will often say things that I later regret |  |
| 8 | J'achève ce que je commence |  | ^e^ I finish what I start |  |
| 9 (R) | J'éprouve du plaisir à prendre des risques |  | ^d^ I quite enjoy taking risks |  |
| 10 (R) | Quand je suis ravi(e), je ne peux pas m’empêcher de m’emballer. |  | ^b^ When overjoyed, I feel like I can’t stop myself from going overboard |  |
| 11 | Une fois que je commence un projet, je le termine presque toujours. |  | ^e^ Once I start a project, I almost always finish it |  |
| 12 (R) | J’aggrave souvent les choses parce que j’agis sans réfléchir quand je suis contrarié(e). |  | ^c^ I often make matters worse because I act without thinking when I am upset |  |
| 13 | D'habitude je me décide après un raisonnement bien mûri |  | ^a^ I usually make up my mind athrough careful reasoning. |  |
| 14 (R) | Je cherche généralement des expériences et des sensations nouvelles et excitantes. |  | ^d^ I generally seek new and exciting experiences and sensations |  |
| 15 (R) | Quand je suis vraiment enthousiaste, j’agis souvent sans réfléchir. |  | ^b^ I tend to act without thinking when I am really excited. |  |
| 16 | Je suis une personne productive qui termine toujours son travail. |  | ^e^ I am a productive person who always gets the job done. |  |
| 17 (R) | Quand je me sens rejeté(e), je dis souvent des choses que je regrette ensuite. |  | ^c^ When I feel rejected, I will often say things that I later regret. |  |
| 18 (R) | Je me réjouis des expériences et sensations nouvelles même si elles sont un peu effrayantes et non conformistes. |  | ^d^ I welcome new and exciting experiences and sensations, even if they are a little frightening and unconventional |  |
| 19 | Avant de me décider, je considère tous les avantages et inconvénients |  | ^a^ Before making up my mind, I consider all the advantages and disadvantages |  |
| 20 (R) | Quand je suis très heureux/heureuse, j’ai l’impression qu’il est normal de céder à ses envies ou de se laisser aller à des excès. |  | ^b^ When I am very happy, I feel like it is OK to give in to cravings or overindulge |  |

# Metacognitions

|  | Traduction | Gierski F, Spada M, Fois E, Picard A, Naassila M, Van der Linden M. (2015) Positive and negative metacognitions about alcohol use among university students: Psychometric properties of the PAMS and NAMS French versions. *Drug and Alcohol Dependence* **153**: 78-85. doi: [10.1016/j.drugalcdep.2015.06.003](https://doi.org/10.1016/j.drugalcdep.2015.06.003) | | |
| --- | --- | --- | --- | --- |
| PAMS | | | | |
|  |  | Two factor model : | *- Emotionnal  - Cognitive |  |
| 1 | Boire me rend plus affectueux(se). | De 1 (pas du tout d’accord)  à  4 (totalement d’accord) | *Drinking makes me more affectionate | 1 (Do not agree)  to  4 (Agree very much) |
| 2 | Boire me rend plus confiant(e) |  | *Drinking makes me more confident |  |
| 3 | Boire me fait penser plus clairement. |  | Drinking makes me think more clearly |  |
| 4 | Boire me fait me sentir plus détendu(e). |  | *Drinking makes me feel more relaxed |  |
| 5 | Boire m’aide à contrôler mes pensées |  | Drinking helps me to control my thoughts |  |
| 6 | Boire rend mes idées noires plus supportables. |  | Drinking makes my negative thoughts more bearable |  |
| 7 | Boire réduit mon anxiété |  | *Drinking reduces my anxious feelings |  |
| 8 | Boire me rend plus sociable |  | *Drinking makes me more sociable |  |
| 9 | Boire me rend moins gêné(e) |  | *Drinking reduces my self-consciousness |  |
| 10 | Boire me fait sentir heureux(se). |  | *Drinking makes me feel happy |  |
| 11 | Boire m’aide à me concentrer. |  | Drinking helps me focus my mind |  |
| 12 | Boire m’aide à m’intégrer socialement |  | *Drinking helps me fit in socially |  |
| 13 | Boire me rend… |  |  |  |
|  | | | | |
| NAMS | | | | |
|  |  | Two factor model : | - Cognitive harm  *- Uncontrollability |  |
| 1 | Je n’ai aucun contrôle sur ma consommation d’alcool. | De 1 (pas du tout d’accord)  à  4 (totalement d’accord) | *I have no control over my drinking | 1 (Do not agree)  to  4 (Agree very much) |
| 2 | Si je n'arrive pas à contrôler ma consommation d’alcool, je vais en mourir. |  | If I cannot control my drinking I will cease to function |  |
| 3 | Boire endommagera mon cerveau. |  | Drinking will damage my mind |  |
| 4 | Ma consommation d’alcool persiste, quel que soit l'effort que je fais pour la contrôler. |  | *My drinking persists no matter how I try to control it |  |
| 5 | Boire me fera perdre le contrôle. |  | Drinking will make me lose control |  |
| 6 | Le fait de boire contrôle ma vie |  | *Drinking controls my life |  |

# Interindividual factors

| DMQ-R Short Form | | | | |
| --- | --- | --- | --- | --- |
|  | English version | Kuntsche E, Kuntsche S. (2009) Development and validation of the drinking motive questionnaire revised short form (DMQ–R SF). *Journal of Clinical Child & Adolescent Psychology* **38**:899-908. doi: [10.1037/t17250-000](https://doi.org/10.1037/t17250-000) | | |
|  |  | 4 factors model : | a – Enhancement  b – Social  c – Conformity  d - Coping |  |
|  | Voici une liste de raisons que les gens donnent pour boire de l’alcool ou consommer des substances psychoactives (cannabis, cocaïne, héroïne, médicaments hors prescription).  Si tu penses à toutes les fois où tu as bu de l'alcool ou consommé des substances psychoactives au cours des 12 derniers mois, combien de fois l'as-tu fait… |  | In the last 12 months, how often did you drink ... |  |
| 1 | pour mieux apprécier une fête | Jamais (1 pt)  Rarement (2 pts)  Parfois (3 pts)  Souvent (4 pts)  Toujours (5 pts) | ^b^because it helps you enjoy a party? | Never  Rarely  Sometimes  Often  Almost always |
| 2 | parce que cela t'aide lorsque tu es déprimé(e) ou nerveux/se |  | ^d^because it helps you when you feel depressed or nervous? |  |
| 3 | pour te réconforter lorsque tu es de mauvaise humeur |  | ^d^to cheer up when you’re in a bad mood? |  |
| 4 | parce que tu aimes les sensations que cela procure |  | ^a^because you like the feeling? |  |
| 5 | pour te soûler |  | ^a^to get high? |  |
| 6 | parce que c'est plus drôle lorsque tu es avec les autres |  | ^b^because it makes social gatherings more fun? |  |
| 7 | parce que tu aimerais faire partie d'un certain groupe |  | ^c^to fit in with a group you like? |  |
| 8 | pour que les fêtes soient plus réussies |  | ^b^because it improves parties and celebrations? |  |
| 9 | pour oublier tes problèmes |  | ^d^to forget about your problems? |  |
| 10 | simplement parce que cela t'amuse |  | ^a^because it’s fun? |  |
| 11 | pour être apprécié(e) par les autres |  | ^c^to be liked? |  |
| 12 | pour ne pas me sentir exclu(e) |  | ^c^so you won’t feel left out? |  |

| Subjective norm | | | | |
| --- | --- | --- | --- | --- |
|  | Inspired from | Ajzen (1991) The theory of planned behavior. *Organizational Behavior and Human Decision Processes* **50**: 179-211. doi: [10.1016/0749-5978(91)90020-t](https://doi.org/10.1016/0749-5978(91)90020-t) | | |
| 1 | La plupart des personnes qui sont importantes pour moi pensent que je devrais consommer de l’alcool pour être défoncé(e) | De 1 (pas du tout d’accord)  à  7 (totalement d’accord) | Most people who are important to me think I should drink alcohol to get high. | 1 (Do not agree)  to  7 (Agree very much) |
| 2 | La plupart des personnes qui sont importantes pour moi me recommandent de consommer de l’alcool pour être défoncé(e) |  | Most people who are important to me recommend that I drink alcohol to get high. |  |
| 3 | Parmi les 5 personnes que vous connaissez le mieux, combien consomment de l’alcool pour se défoncer ? |  | Among the 5 people you know best, how many of them drink alcohol to get high? |  |
| 4 | La plupart des personnes qui sont importantes pour moi consomment de l’alcool pour être défoncées |  | Most people who are important to me drink alcohol to get high |  |
| Drinking identity | | | | |
|  | Inspired from | Callero P. (1985) Role-Identity Salience. *Social Psychology Quarterly* **48**: 203. doi: [10.2307/3033681](https://doi.org/10.2307/3033681) | | |
| 1 | Le fait de consommer excessivement de l’alcool est une part importante de qui je suis. | De 1 (pas du tout d’accord)  à  7 (totalement d’accord) | Drinking excessively is an important part of who I am. | 1 (Do not agree)  to  7 (Agree very much) |
| 2 | Je me considère comme une personne qui consomme excessivement de l’alcool |  | I am like the kind of person who drinks excessively |  |
