# Supplementary material for: Predictors of alcohol use disorder risk in young adults: Direct and indirect psychological paths through binge drinking
Source: PLoS One. 2025 May 9;20(5):e0321974. doi: 10.1371/journal.pone.0321974 (PMC12064033; doi:10.1371/journal.pone.0321974)
Supplement: Attachment — Submitted filename: Comment - Plos one article.docx [file pone.0321974.s002.docx]

**Comment 1**: Indicate the number of participants with missing data for each variable of interest

**Comment 2**: Although the introduction lists psychological determinants like drinking motives and personality traits, there is minimal operational definition of these constructs.

For instance, "impulsivity" or "drinking identity" could be elaborated upon to provide readers a better grasp of their theoretical and practical implications within this model.

**Comment 3**: The choice to focus on three AUDIT subdimensions—alcohol intake, dependence symptoms, and alcohol-related problems—is central to the study, yet the rationale for this distinction could be clearer. Further expounding on how these subdimensions uniquely relate to AUD risk and why it is important to differentiate them would strengthen the study's conceptual framework.

**Comment 4**: Although the dual-pathway model proposed is intriguing, a more detailed outline of the hypothesized mechanisms for each pathway (direct vs. indirect) would clarify the expected relationships. For instance, it is unclear if and how specific intra-individual factors might interact with inter-individual ones or if the pathways are entirely independent.

**Comment 5**: The introduction suggests that inter-individual factors, such as drinking norms, are more predictive of BD but not necessarily AUD. However, given that social influences often persist in university environments and influence other subdimensions of AUD, such as alcohol-related problems, this assertion may warrant further support or nuanced discussion.

**Comment 6**: While several studies are referenced, a broader citation base for the dual-pathway model's antecedents, including existing models that address AUD progression, would improve the literature review. Including models that also segment AUD risk factors in a population-specific context (e.g., young adults or university students) could also enhance relevance.

**Comment 7**: The generalisability (external validity) of the study results could be explained more

**Comment 8**: The introduction briefly mentions that the findings could inform prevention strategies, but this point could be expanded. What types of interventions might be designed based on a dual-pathway model? Addressing this could provide a more immediate real-world application of the study’s anticipated findings.
