# Supplementary material for: Predictors of alcohol use disorder risk in young adults: Direct and indirect psychological paths through binge drinking
Source: PLoS One. 2025 May 9;20(5):e0321974. doi: 10.1371/journal.pone.0321974 (PMC12064033; doi:10.1371/journal.pone.0321974)
Supplement: Attachment — Submitted filename: Response to Reviewers.docx [file pone.0321974.s003.docx]

Dear Editor, Dear Reviewers,

We greatly appreciate your interest for our manuscript, as well as your feedback and comments. We have fully addressed all your comments, leading to an improved version of the manuscript. Please find below our specific response to each comment and the changes made to the revised manuscript (in red).

We thank you for your input and hope you will be satisfied with our responses and modifications.

Yours sincerely,

The authors.

# **Editor**

### The study is well conceived and conducted.

We thank the Editor for this very positive evaluation of our work.

### The non-response rate is on the higher side. Is there any effort made to assess if there is a systemic difference between responders and non-responders?

First of all, our response rate (14.2%) is close to or even higher than those reported in previous studies targeting University students, a population known to produce limited response rates (Ehret et al., 2013; Lannoy et al., 2020; Mauduy et al., 2022; Tavolacci et al., 2013). The key question is however whether our sample is representative of the population of students at Caen Normandie University, and beyond of the general population of University students in France and Europe. Our complementary analyses prove that our sample is indeed representative. More specifically, we examined our sample of respondents (*N* = 3983) with regard to three characteristics: their socio-demographic characteristics (gender, and level of study), their characteristics in terms of alcohol consumption (alcohol consumption, BD) and their characteristics in terms of psychopathological variables (depression and anxiety). We then compared them with alcohol consumption in France, as well as with other samples of students in France and Europe (e.g., Germany, Belgium).

Socio-demographic variables

In France and Europe, the university population is broadly uniform in terms of age (18-25 years), gender (55-60% women), and level of study (around 65% undergraduate students). Our sample (*M*_age_ = 20.46, 63% of women and 74% of undergraduates) is consistent with these data, as are previous studies on alcohol consumption among students (e.g., Ehret et al., 2013; Lannoy et al., 2020; Bollen et al., 2021; Mange et al., 2021). For example, Tovalacci et al. (2013) reported 63.4% women and 80.2% undergraduates.

Alcohol consumption variables

With regard to the overall AUDIT score, our sample (average 7.58) appears to be in line with other samples in France and Europe. In France, for example, one study obtained scores comparable to ours from three samples of French students (6.99; 7.28; 7.65; Gierski et al., 2015). In addition, a study of young French adults (aged 18-25) also obtained an overall AUDIT score comparable to ours (i.e., 6.44; Bakkali et al., 2023). Furthermore, compared with samples of students in Europe, the overall AUDIT score of our sample appears to be average: Belgium (9.19; Bollen et al., 2021), Denmark (9.52; Cooke et al, 2019), England (9.99; Cooke et al., 2019), Germany (5.44; Cooke et al., 2019), Italy (5.55; Cooke et al., 2019), Portugal (4.90; Cooke et al., 2019), Switzerland (5.62; Cooke et al., 2019).

Psychopathological variables (depression and anxiety)

Our total sample had a mean depression score of 6.96 and a mean anxiety score of 48.10. These mean scores, indicating mild levels of depression and anxiety in our sample, are comparable to those obtained in other samples of young people in France (Bakkali et al., 2023; Gierski et al, 2015) and Europe (Portugal, Andrade et al., 2001; Belgium, De Coninck et al., 2021; Germany, Zunhammer et al., 2008; Spain, Gijón Puerta et al., 2022; Germany, Denmark, Poland, Bulgary, Mikolajczyk et al., 2008).

**Main changes made to the manuscript**

- In the *Procedure and participants* (*Method*) section, we added the information that our sample was comparable to those of previous studies in France and Europe exploring alcohol consumption among students (p. 11)

“Our sample is comparable in terms of socio-demographic data, alcohol consumption and psychopathological variables to those of previous studies exploring alcohol consumption among European university students^1,26,28,70–75^”

### If there is a diagrammatic representation of the final pathway model, it will add to comprehensibility of the study.

Following this insightful comment, we now propose in our new Figure 3 a diagrammatic representation of the final pathway model. This figure shows that the risk of AUD, and particularly the dimensions linked to alcohol dependence and alcohol problems:

are predicted by a direct psychological pathway consisting mainly of intra-individual factors (factors in blue)

are predicted by an indirect psychological pathway, mediated by the BD. This indirect psychological pathway is made up of known determinants of BD, i.e., all the inter-individual factors measured in this study (factors in orange: conformity motives, social motives, social norms, social identity) and intra-individual factors characteristic of BD use (e.g., sensation-seeking, enhancement motives).

**Fig 3.** **Results of the psychological dual-path model of Risk of Alcohol Use Disorders in University students according to the three AUDIT dimensions**

**3a. Alcohol intake**	**3b. Alcohol dependence symptoms	3c. Alcohol-related problems**

*Note.* Inter-individual factors are in orange. Intra-individual factors are in blue. Transparent factors are those that show no significant association with binge drinking or alcohol-related outcomes. Numbers below factors are standardized coefficients. Numbers in purple are for the link between factors and binge drinking. Numbers in red are for the links between factors and outcomes (alcohol intake, alcohol dependence, and alcohol-related problems). Statistically significant at ^***^*p* < .001; ^**^*p* < .01; ^*^*p* < .05.

**Main changes made to the manuscript**

- In the *Results* section, we have added a figure (Fig. 3) showing the main results of our statistical model (p. 18).

### The comments by the reviewers may be addressed.

We comprehensively addressed all the comments made by both Reviewers.

# **Reviewer 1**

### Comment 1: Indicate the number of participants with missing data for each variable of interest

We have no missing data for our final sample (*N* = 2026 students) included in the statistical analyses. The bootstasp method (N = 10000 resamples) that we used to obtain better estimates of our statistical parameters does not allow us to deal with missing data (for example with the FIML method, Full Information Maximum Likehood). We therefore had to select only those students for whom we could create a score for each of our variables of interest.

Of the 3938 students who responded to our survey, 616 did not respond to the variables related to alcohol consumption and 472 had not consumed alcohol in the last 12 months.

Of the remaining 2850 students:

- 12 did not respond to the BD measurement items and/or
- 11 did not answer the AUDIT measurement items and/or
- 279 did not respond to the PAMS measurement items - Emotional regulation
- 280 did not respond to the PAMS measurement items - Cognitive regulation
- 282 did not respond to the NAMS measurement items - Uncontrollability
- 290 did not respond to items measuring drinking social norms
- 294 did not respond to the items measuring drinking social identity
- 472 did not respond to the DMQR measure - Social motives
- 472 did not respond to the DMQR measure - Coping motives
- 472 did not respond to the DMQR measure - Enhancement motives
- 472 did not respond to the DMQR measure - Conformity motives
- 615 did not respond to the UPPS measure - Negative Urgency
- 611 did not respond to UPPS - Positive Urgency items
- 609 did not answer the UPPS - Lack of premeditation questions
- 614 did not respond to the UPPS measure Perseverance
- 614 did not respond to the UPPS measure Sensation seeking
- 670 did not respond to the STAI-T measure - Anxiety
- 744 did not respond to the BDI measure - Depression symptoms

Thus, 824 participants were excluded because their answers to at least one of the variables of interest above were missing. In the end, our final analyzed sample consisted of 2026 participants. To summarize this information, we have added the flow diagram below to our revised manuscript.

**Fig 2. Flow diagram**

**Main changes made to the manuscript**

- In the *Procedure and participants* (*Method*) section, we have added a figure (Fig. 2) representing the selection process for our final sample (p. 11)
- In the *Statistical Analysis* (*Method*) section, we have indicated that we have no missing data in our statistical model (p. 16).

“As the bootstrapping approach does not allow to deal with missing data, our statistical model carried out on our sample of 2026 students did not include any missing data.”

### Comment 2: Although the introduction lists psychological determinants like drinking motives and personality traits, there is minimal operational definition of these constructs.

### For instance, "impulsivity" or "drinking identity" could be elaborated upon to provide readers a better grasp of their theoretical and practical implications within this model.

We would like to thank you for your comments, which have enabled us to improve the presentation of the psychological factors studied in this research and to further develop the rationale of the dual pathway model. In doing so, we have made several changes to our *Introduction* section.

First, in the subsection “*Inter- and intra-individual psychological factors associated with AUD risk*”, we have further defined each psychological factor studied in our research and given a brief description of its role in alcohol use disorders.

Second, in the following sections “*An indirect psychological pathway from BD to AUD*” and “*A direct psychological pathway to AUD risk*”, we have further justified the role of these psychological factors in the dual path model.

Below are the main changes made to our *Introduction* section.

**Main changes made to the manuscript**

- In the *Inter- and intra-individual psychological factors associated with AUD risk* (*Introduction*) section, we further defined the psychological factors studied in this research and gave a brief description of their role in alcohol use disorders (p. 4-5).

“A substantial body of research has identified psychological factors associated with AUD risk in university students, which can be broadly categorized as either inter-individual or intra-individual^12,13^.

Three major inter-individual factors have consistently been linked to AUD risk in young populations. First, *drinking social identity* - the extent to which individuals identify themselves as part of the social category of “drinkers”^14,15^ - positively predicts alcohol use and AUD risk among youth^15–18^. Second, based on Cooper’s motivational model^19^, two external drinking motives, namely *social* (i.e., positive external motives such as drinking to enhance social interactions) and *conformity* (i.e., negative external motives such as drinking to avoid social censure or rejection) *motives* have emerged as strong predictors of alcohol use^16,20^ and AUD risk^19,21–23^. Notably, higher levels of social motives are positively associated with AUD risk, while conformity motives are negatively related to AUD risk. Third, *drinking social norms* - individuals' beliefs about the acceptability and prevalence of alcohol use among peers^24^, influence alcohol use^25^ and AUD risk^26^. Specifically, more positive perceptions of peer drinking are associated with alcohol consumption and increased AUD risk.

Three key intra-individual factors also contribute to AUD risk in young people. First, according to Cooper’s motivational model, two internal drinking motives, namely *enhancement* (i.e., positive internal motive such as drinking to elevate positive mood) and *coping* (i.e., negative internal motives such as drinking to regulate negative affect) *motives* are associated with greater alcohol use and AUD risk in young people^12,16,20,27^. Second, *alcohol-related metacognitions* - schematic information that individuals hold about the significance of their cognitive experience and ways to control it - are significant predictors of AUD risk^28,29^. These metacognitions include positive beliefs about the impact of alcohol use on *cognitive* (helping to control thoughts) and *emotional* (helping to improve mood) *self-regulation*, and negative metacognitions related to the perceived *uncontrollability* and the *negative cognitive functioning impact* of alcohol use. Third, *personality traits and psychopathological variables*, such as loneliness (i.e., a distressing feeling of isolation perception or social rejection^30,31^), anxiety^32^, depression symptoms^33^, and impulsivity (i.e., the tendency to act prematurely without considering consequences) have been consistently associated with alcohol use^34,35^ and AUD risk^31,36,37^. More precisely, impulsivity, as conceptualized in the UPPS model^38^, encompasses dimensions such as *negative* and *positive urgency* (i.e., the tendency to act rashly in intense negative or positive emotional contexts), *lack of perseverance* (i.e., the difficulty to maintain attention on a task), *lack of premeditation* (i.e., the difficulty to plan behaviors and consider their consequences), and *sensation seeking* (i.e., the propensity to look for new and exciting experiences), all of which have been linked to alcohol-related outcomes^39^.

However, it is worth noting that these psychological determinants of AUD risk, and particularly drinking social identity and enhancement motives, are also the strongest predictors of BD^12,26^, suggesting that BD and AUD may share common psychological predictors, that an *indirect psychological pathway* might lead to AUD through BD, and that preventing BD could reduce AUD risk among students.”

- In the section *An indirect psychological pathway from BD to AUD* (*Introduction*), we further justified the place of the psychological factors involved in the indirect pathway to AUD (p. 5-6).

“Social factors are widely recognized as primary initiators of alcohol use in young adults^40–43^, a highly social behavior^44^, characterized by the BD practice, a socially motivated drinking pattern^45^. Indeed, theoretical models, such as Koob and Volkow’s three-stage conceptualization of addiction^46^ and the socioecological framework of drinking contexts^47^, emphasize that excessive alcohol consumption, and particularly BD, is a critical precursor to AUD development. Consistent with these models, research shows that BD is a significant risk factor for AUD^6,7,48^.

BD, as a social drinking pattern, is primarily driven by psychological factors that can be classified into inter-individual (e.g., drinking social identity, drinking norms, social motives) and intra-individual (e.g., positive enhancement motives, sensation seeking) categories. These factors align with the positive affect regulation theory of AUD etiology, which conceptualizes AUD as a maladaptive strategy to enhance positive emotions and social experiences^40^. According to this theory, positive alcohol expectancies - beliefs about alcohol’s ability to improve emotional states and facilitate social bonding - encourage individuals to consume alcohol and increase the likelihood of developing AUD. Supporting this perspective, studies have demonstrated that these BD-related psychological factors, especially interpersonal ones, are indirectly linked to AUD symptoms through higher levels of alcohol consumption^21,49,50^.

Consequently, rather than exerting a direct influence on AUD risk, we hypothesize that these psychological factors primarily promote BD, which in turn increases AUD risk in young adults. In other words, psychological factors associated with the positive affect regulation conceptualization might lead young adults to engage in BD, which subsequently increases their AUD risk. However, existing studies often fail to account for this indirect pathway - and therefore report direct associations - due to two key methodological limitations.”

- In the section *A direct psychological pathway to AUD* *risk* (*Introduction*), we further justified the role of the psychological factors involved in the direct pathway to AUD (p. 8-9)

“One of the most prominent theories of AUD etiology suggests that AUD develops as a way to alleviate negative affect, such as reducing anxiety, stress, and depression^27,59–62^. Many young adults report drinking to cope with negative emotions^26,63,64^. Psychological factors associated with this negative affect regulation process - such as negative reinforcement motives (i.e., coping motives), negative alcohol-related metacognitions, and specific personality traits - may play a direct role in AUD risk, independent of BD, which would be less influenced by these factors.

Empirical evidence supports this perspective. Studies focusing on the prediction of dependence symptoms and alcohol-related problems - rather than overall AUDIT scores - reveal notable differences between psychological predictors of BD and those of AUD risk.

Regarding drinking motives, while BD is primarily explained by social and enhancement motives, coping motives are more strongly associated with alcohol-related problems^27,65^ and dependence symptoms^32,66^. Thus, coping motives appear to exert a direct influence on AUD risk, independent of BD practice.

Regarding alcohol-related metacognitions, empirical research highlights distinct roles for positive and negative metacognitions. Positive metacognitions, such as emotional self-regulation, are primarily associated with BD^28^. Conversely, negative metacognitions (e.g., beliefs about uncontrollability and negative consequences of alcohol use) are crucial for the persistence of alcohol consumption^67,68^ and are significant predictors of alcohol dependence ^69^. These findings suggest that negative metacognitions play a direct role in AUD risk among university students, independent of BD practice.

Regarding personality traits, the predictive role of impulsivity dimensions differs between alcohol use and alcohol-related problems or dependence symptoms. While the lack of perseverance is more strongly associated with alcohol use, negative urgency and the lack of premeditation are more predictive of AUD risk^39^. Additionally, loneliness is a characteristic specific to higher AUD risk compared to lower dependence risk^31^, and depression symptoms have been more strongly associated with alcohol dependence symptoms than with general alcohol use^33^. These findings indicate that some impulsivity dimensions (e.g., negative urgency and lack of premeditation), as well as loneliness and depression, may directly contribute to AUD risk among university students, beyond their association with BD.”

### Comment 3: The choice to focus on three AUDIT subdimensions—alcohol intake, dependence symptoms, and alcohol-related problems—is central to the study, yet the rationale for this distinction could be clearer. Further expounding on how these subdimensions uniquely relate to AUD risk and why it is important to differentiate them would strengthen the study's conceptual framework.

We have now better justified our decision to focus on the AUDIT sub-dimensions rather than the overall AUDIT score.

Most studies predicting AUD risk among university students use the total score of the AUDIT as the outcome variable. However, according to the original authors, AUDIT is not a unitary scale as it encompassed three major dimensions associated with AUD risk: alcohol intake, alcohol dependence symptoms, and alcohol-related problems (Saunders et al., 1993). These three sub-dimensions (i) correspond to different aspects of alcohol consumption (Saunders et al., 1993; Gache et al., 2005; Horváth et al., 2023; Babor & Robaina, 2016), (ii) have different predictors and causal factors (Simons et al., 2009), and (iii) are themselves different predictors of drinking behaviour and its consequences (Conigrave et al., 1995). Notably, the alcohol intake dimension is highly correlated with BD, as evidenced by the sensitivity and specificity of AUDIT-C in detecting BD (García Carretero et al., 2016; Tomás et al., 2017). In contrast, the other two dimensions - dependence symptoms and alcohol-related problems - capture distinct aspects of AUD risk from excessive consumption (Horváth et al., 2023; Babor et al., 2016). Consequently, studies relying on the overall AUDIT score may overestimate the role of BD-related psychological determinants in AUD risk and underestimate factors more closely tied to dependence symptoms and alcohol-related problems. Therefore, a growing body of evidence suggests that the AUDIT should be distinguished according to its sub-dimensions to investigate the risk of AUD (Babor & Robaina, 2016; Gecaite-Stonciene et al., 2021; Horváth et al., 2023; Moehring et al., 2018; Peng et al., 2011). This is also confirmed by our data. Through confirmatory factor analyses testing the dimensionality of the AUDIT, we checked that this distinction between three sub-dimensions offered the best fit with our own data, compared to an overall AUDIT score. While the model comprising a single dimension (overall AUDIT score) showed a poor fit to the data (χ²_(35)_ = 1128, *p* < .001, CFI = .852, TLI = .810, RMSEA = .105 , 90% CI [.10, .11], SRMR = .049), the model distinguishing the three AUDIT sub-dimensions showed an excellent fit to the data (χ²_(32)_ = 383, *p* < .001, CFI = .953, TLI = .933, RMSEA = .062 , 90% CI [.057, .068], SRMR = .035). This reinforces the idea of going beyond the overall AUDIT score, to consider each of the AUDIT dimensions separately to investigate AUD risk.

We have added these elements to the revised manuscript.

**Main changes made to the manuscript**

- In the section *Methodological limitations affecting the understanding of AUD risk* (*Introduction*), we have added elements justifying our interest in the three AUDIT sub-dimensions rather than in the overall AUDIT score (p. 6-8)

“First, research investigating psychological determinants of AUD typically examines these factors in isolation from BD, focusing on their direct effects on AUD risk without considering the mediating role of BD. This approach overlooks the possibility that BD may serve as a mediator between some psychological factors (e.g., drinking identity, social motives) and AUD risk^21,50^, potentially explaining why many determinants appear common to both BD and AUD risk.

Second, most studies predicting AUD risk among university students use the overall AUDIT score as the outcome variable. According to the original authors, AUDIT measures three major dimensions associated with AUD risk: alcohol intake, alcohol dependence symptoms, and alcohol-related problems^51^. However, these three sub-dimensions (i) correspond to different aspects of alcohol consumption^9,10,51,52^, (ii) have different predictors and causal factors^53^, and (iii) are themselves different predictors of drinking behavior and its consequences^54^. Notably, the “alcohol intake” dimension is highly correlated with BD, as evidenced by the sensitivity and specificity of AUDIT-C in detecting BD^55,56^. In contrast, the other two dimensions - dependence symptoms and alcohol-related problems - capture distinct aspects of AUD risk from excessive consumption^9,10^. Consequently, studies relying on the overall AUDIT score may overestimate the role of BD-related psychological determinants in AUD risk and underestimate factors more closely tied to dependence symptoms and alcohol-related problems. Therefore, a growing body of evidence suggests that the AUDIT is not a unitary scale but should rather be distinguished according to its sub-dimensions to investigate the risk of AUD^9–11,57,58^.

Thus, these two methodological issues may lead to (i) an overestimation of AUD predictors that are shared with BD but primarily act indirectly through BD, and (ii) an underestimation of predictors less correlated with BD but directly influencing AUD risk. Indeed, when focusing specifically on dependence symptoms and alcohol-related problems, some evidence suggests that specific psychological factors are associated with AUD risk among youth, highlighting a direct psychological pathway to AUD, independent from BD.”

- In the *Measures* (*Method*) section, we have added the results of our confirmatory factor analyses testing the dimensionality of the AUDIT. (p. 13-14)

“We measured risk of AUD from the Alcohol Use Disorders Identification Test (AUDIT^52^). The AUDIT is a 10-item measure designed to identify individuals at risk for AUD by assessing three subdimensions^52,66^: *alcohol intake* (items 1-3, frequency of drinking, number of drinks consumed on a typical day, frequency of heavy drinking), *alcohol dependence symptoms* (items 4-6, impaired control over drinking, increased salience of drinking, morning drinking), and *alcohol related-problems* (items 7-10, guilt after drinking, blackouts, alcohol-related injuries, others concerned about drinking). We tested the dimensionality of the AUDIT using confirmatory factor analyses. While the model comprising a single dimension (overall AUDIT score) showed a poor fit to the data (χ²_(35)_ = 1128, *p* < .001, CFI = .852, TLI = .810, RMSEA = .105 , 90% CI [.10, .11], SRMR = .049), the model distinguishing the three AUDIT sub-dimensions showed an excellent fit to the data (χ²_(32)_ = 383, *p* < .001, CFI = .953, TLI = .933, RMSEA = .062 , 90% CI [.057, .068], SRMR = .035). This reinforces the idea of avoiding the use of an overall AUDIT score to rather measure each of the three AUDIT subdimensions separately to investigate AUD risk. Thus, we computed a score for alcohol intake (α = 0.73), a score for alcohol dependence symptoms (α = 0.81), and a score for alcohol-related problems (α = 0.76).”

### Comment 4: Although the dual-pathway model proposed is intriguing, a more detailed outline of the hypothesized mechanisms for each pathway (direct vs. indirect) would clarify the expected relationships. For instance, it is unclear if and how specific intra-individual factors might interact with inter-individual ones or if the pathways are entirely independent.

We thank you for your comment, which has enabled us to look more closely at the psychological mechanisms involved in each of the pathways in the *Introduction* section and then to discuss them further in the *Discussion* section.

Regarding the indirect path to AUD risk via BD, we know that theoretical models, such as Koob and Volkow’s three-stage conceptualization of addiction (2010) and the socioecological framework of drinking contexts (Freisthler et al., 2014), emphasize that excessive episodic alcohol consumption is a critical precursor to AUD development. Consistent with these models, research shows that BD is a significant risk factor for AUD (Nietyo et al., 2021; Patrick et al., 2011; Tavolacci et al., 2019). Further, BD, as a social drinking pattern, is primarily driven by psychological factors that can be classified into inter-individual (e.g., drinking social identity, drinking norms, social motives) and intra-individual (e.g., positive enhancement motives, sensation seeking) categories. These factors align with the positive affect regulation theory of AUD etiology, which conceptualizes AUD as a maladaptive strategy to enhance positive emotions and social experiences (Cooper et al., 2015). According to this theory, positive alcohol expectancies - beliefs about alcohol’s ability to improve emotional states and facilitate social bonding - encourage individuals to consume alcohol and increase the likelihood of developing AUD. Supporting this perspective, studies have demonstrated that these BD-related psychological factors, especially those of a social nature, are indirectly linked to AUD symptoms through higher levels of alcohol consumption (Magid et al., 2007; Reed et al., 2007; Simons & Carey, 2006). Consequently, rather than exerting a direct influence on AUD risk, we hypothesize that these psychological determinants primarily promote BD, which in turn increases AUD risk. In other words, psychological factors associated with the positive affect regulation conceptualization lead young adults to engage in BD, which subsequently increases their AUD risk.

Regarding the direct path to AUD risk, one of the most prominent theories of AUD etiology suggests that AUD develops as a way to alleviate negative affect, such as reducing anxiety, stress, and depression (Baker et al., 2004; Greeley & Oei, 1999; Khantzian, 2017; Kuntsche et al., 2006; Skrzynski & Creswell, 2020). Many young adults report drinking to cope with negative emotions (Bakkali et al., 2023; Cooper et al., 1995; Sher et al., 2011). Psychological factors associated with this negative affect regulation process - such as negative reinforcement motives (i.e., coping motives), negative alcohol-related metacognitions, and specific personality traits - may play a direct role in AUD risk, independent of BD practice.

Furthermore, our statistical centered-variables approach, rather than centered-individual approach, seems to indicate that the two pathways can coexist within the same individual. Moreover, this dual pathway is consistent with a recent conceptualization of AUD risk in young people. According to Creswell (2021), a first pathway to AUD risk in young people would involve social drinking and its social determinants, and a second pathway, less linked to heavy drinking but more to solitary drinking, would involve rather intra-individual determinants associated with the negative affect regulation process. According to this conceptualization, these two pathways can also coexist in the same person, particularly when a person who drinks mainly alone as a result of negative reinforcement may also drink socially.

Finally, our research aimed to explore the major pathways of factors that predict AUD risk, by demonstrating the existence of these two pathways and the need to take them into account separately because of their distinct determinants. Although intra-individual and inter-individual factors may interact together to predict BD or AUD risk - notably because each of these two behaviors is always influenced by a specific combination of inter-individual and intra-individual factors (e.g., drinking social identity, drinking norms, social motives and positive enhancement motives, sensation seeking for BD) - clarifying the interactions between these two types of factors within each pathway would be an interesting research perspective to pursue, in particular via the use of cluster or latent profile analyses.

**Main changes made to the manuscript**

- In the *Introduction* section, we have added information on the psychological mechanisms involved in each of the pathways (p. 5-6 and p. 8-9)
- In the *Discussion* section, we have added some elements for discussion on this dual pathway model towards AUD (p. 24-25)

“Our results thus support the dual-pathway model to AUD risk in university students, with an indirect pathway via BD, primarily consisting of interindividual factors and accounting for 37.12% of alcohol intake, 20% of dependence symptoms, and 22.61% of alcohol-related problems, and a direct pathway, primarily consisting of intraindividual factors. This is in line with a recent conceptualization of AUD risk in young people which also considers two paths to AUD^96^. The first pathway to AUD in young people would involve the social practice of alcohol consumption, predicted by psychosocial factors linked to the idea of alcohol consumption for positive reinforcement purposes. A second pathway to AUD in young people would involve solitary drinking, predicted by intra-individual psychological factors associated with the idea of drinking for negative reinforcement.

Therefore, future studies should further explore our dual pathway, particularly to identity the drinking patterns of youth who follow the direct pathway to AUD risk. Do they tend to drink alone? Additionally, it is important to determine whether these two pathways are mutually exclusive or whether they can coexist within the same individual. While our variable-centered statistical approach demonstrates the presence of both pathways as distinct and independent - each operating beyond the influence of the other - it also suggests that they may coexist. Supporting this notion, recent research suggests that these two pathways might operate simultaneously within individual. Youth solitary drinkers, who could instead take the direct path to AUD, still spend the majority of their time drinking in social settings^97,98^.”

- In the *Discussion* section, we have mentioned the interesting research perspective aimed at examining the interactions between intra- and inter-individual factors within each of the two paths (p. 26)

“Finally, although our research aimed to explore the major pathways of psychological factors that predict AUD risk, intra-individual and inter-individual factors may interact together to predict BD or AUD risk notably because each of these two alcohol outcomes is always influenced by a specific combination of inter-individual and intra-individual factors. Therefore, clarifying the interactions between these two types of factors within each pathway would be an interesting research perspective to pursue by using cluster or latent profile analyses.”

### Comment 5: The introduction suggests that inter-individual factors, such as drinking norms, are more predictive of BD but not necessarily AUD. However, given that social influences often persist in university environments and influence other subdimensions of AUD, such as alcohol-related problems, this assertion may warrant further support or nuanced discussion.

With this research, we do not challenge the idea that social factors (in particular social norms) play a major role in AUD and particularly in alcohol-related problems. Indeed, numerous studies have shown that social norms influence the negative consequences of alcohol consumption (e.g., Lee et al. 2010; Wood et al., 1992) and that designing prevention measures targeting social norms allows to reduce these negative consequences (e.g., DeJong, 2010). This being said, this research tends to suggest that the influence of social norms relates more to alcohol consumption (and BD) than to the symptoms of dependence and the negative consequences associated with this consumption (Neighbors et al., 2008). Social factors, as the first step towards alcohol consumption by young people (Cooper et al., 2016; Maggs & Schulenberg, 2005; Mundt, 2011; Trucci, 2020), would lead them to consume heavily alcohol in social situations as observed in BD, a practice which would lead to the occurrence of negative consequences. The risk of AUD would emerge in young people because their normative social environment leads them to practice BD, as part of the indirect psychological pathway to AUD. As a whole, while we surely acknowledge that inter-personal factors play a predicting role in the whole continuum of AUD, we wanted to underline that it is a specific risk factor for BD, as further proven by our data.

We have therefore clarified our point and added these elements to the discussion of the revised manuscript.

**Main changes made to the manuscript**

- In the *Discussion* section, we have added discussion elements indicating that this research does not call into question the influence of social norms on severe alcohol use disorder, but that it suggests that the influence of norms relates more to the consumption of alcohol and its initiation in young people, than to the symptoms of dependence and the negative consequences associated with this consumption (p. 24).

“This research results do not challenge the notion that social factors, particularly social norms, play a significant role in AUD and alcohol-related problems. Extensive evidence demonstrates that social norms influence the negative consequences of alcohol consumption^99,100^ and that prevention strategies targeting these norms can effectively reduce such consequences^101^. However, our results rather suggest that the influence of social norms may be more directly related to excessive alcohol consumption behaviors (i.e., BD) than to dependence symptoms or the associated negative outcomes, consistently with some previous studies^47,102^. Social factors may serve as a gateway to alcohol consumption among young people, encouraging heavy social drinking behaviors such as BD, which in turn lead to negative consequences. The risk of AUD among young people appears to arise indirectly, as their normative social environment promotes BD, thereby contributing to the emergence of dependence symptoms and alcohol-related problems along this indirect psychological pathway.”

### Comment 6: While several studies are referenced, a broader citation base for the dual-pathway model's antecedents, including existing models that address AUD progression, would improve the literature review. Including models that also segment AUD risk factors in a population-specific context (e.g., young adults or university students) could also enhance relevance.

As indicated previously in our response of your comment 4, we have now provided more information about the existing models (i.e., Koob and Volkow’s three-stage conceptualization of addiction, the socioecological framework of drinking contexts, positive affect regulation theory, negative affect regulation theory, the Social-contextual framework of risk for alcohol use disorder, ) that address AUD progression.

**Main changes made to the manuscript**

- In the *Introduction* and *Discussion* sections, we have provided more information about the existing models that address AUD progression. (p. 5, 8, and 24-25).

“Social factors are widely recognized as primary initiators of alcohol use in young adults^40–43^, a highly social behavior^44^, characterized by the BD practice, a socially motivated drinking pattern^45^. Indeed, theoretical models, such as Koob and Volkow’s three-stage conceptualization of addiction^46^ and the socioecological framework of drinking contexts^47^, emphasize that excessive alcohol consumption, and particularly BD, is a critical precursor to AUD development. Consistent with these models, research shows that BD is a significant risk factor for AUD^6,7,48^.

BD, as a social drinking pattern, is primarily driven by psychological factors that can be classified into inter-individual (e.g., drinking social identity, drinking norms, social motives) and intra-individual (e.g., positive enhancement motives, sensation seeking) categories. These factors align with the positive affect regulation theory of AUD etiology, which conceptualizes AUD as a maladaptive strategy to enhance positive emotions and social experiences^40^. According to this theory, positive alcohol expectancies - beliefs about alcohol’s ability to improve emotional states and facilitate social bonding - encourage individuals to consume alcohol and increase the likelihood of developing AUD. Supporting this perspective, studies have demonstrated that these BD-related psychological factors, especially interpersonal ones, are indirectly linked to AUD symptoms through higher levels of alcohol consumption^21,49,50^.”

“One of the most prominent theories of AUD etiology suggests that AUD develops as a way to alleviate negative affect, such as reducing anxiety, stress, and depression^27,59–62^. Many young adults report drinking to cope with negative emotions^26,63,64^. Psychological factors associated with this negative affect regulation process - such as negative reinforcement motives (i.e., coping motives), negative alcohol-related metacognitions, and specific personality traits - may play a direct role in AUD risk, independent of BD, which would be less influenced by these factors.”

“Our results thus support the dual-pathway model to AUD risk in university students, with an indirect pathway via BD, primarily consisting of interindividual factors and accounting for 37.12% of alcohol intake, 20% of dependence symptoms, and 22.61% of alcohol-related problems, and a direct pathway, primarily consisting of intraindividual factors. This is in line with a recent conceptualization of AUD risk in young people that also considers two pathways to AUD^103^. The first pathway to AUD in young people would involve the social practice of alcohol consumption, predicted by psychosocial factors linked to the idea of alcohol consumption for positive reinforcement purposes. A second pathway to AUD in young people would involve solitary drinking, predicted by intra-individual psychological factors associated with the idea of drinking for negative reinforcement.”

### Comment 7: The generalisability (external validity) of the study results could be explained more

Although our study focused on a population of young students, the model tested appears to be generalizable to other populations and other contexts, since the variables tested are general determinants of consumption beyond the specific context. Nevertheless, it remains important to test this model on other populations to verify the generalizability of our results.

We have clarified this point in the discussion of the revised manuscript.

**Main changes made to the manuscript**

- In the *Limitations and future directions* (*Discussion*) section, we have added elements clarifying the generalizability of our results (p. 28)

“Finally, while our results are consistent with previous studies^12,66^ and while the model tested appears generalizable to other populations and contexts, beyond university students (since the variables tested are general determinants of alcohol consumption and AUD risk beyond specific contexts), their generalizability should be confirmed in future studies on more various samples.”

### Comment 8: The introduction briefly mentions that the findings could inform prevention strategies, but this point could be expanded. What types of interventions might be designed based on a dual-pathway model? Addressing this could provide a more immediate real-world application of the study’s anticipated findings.

Following this suggestion, we have added a discussion point to our revised manuscript addressing the prevention perspectives arising from this dual pathway model of AUD risk by considering potential prophylactic or therapeutic interventions.

**Main changes made to the manuscript**

- In the *Discussion* section, we have added a sub-section entitled ‘*Practical implications of the dual pathway model of AUD risk*’, addressing the prevention perspectives arising from this dual pathway model (p. 27).

“**Practical implications of the dual pathway model of AUD risk**

Since BD is a highly prevalent drinking pattern among students and a risk factor for developing AUD, practitioners might focus on preventing this drinking behavior to mitigate the risk of AUD. To achieve this, prevention interventions should target the key psychological determinants of BD, and centrally social factors. Interventions addressing social norms through techniques like normative feedback^107^ or induced hypocrisy^108^, as well as interventions targeting drinker social identity using the multi-categorization technique^109^, could prove effective in reducing this excessive alcohol consumption pattern and reduce the transition towards AUD.

However, the findings of this research, particularly the identification of the dual pathway, suggest that BD is not the sole route to AUD risk among young people. Other psychological factors, primarily intra-individual in nature and only weakly linked (or unrelated) to BD, also contribute to AUD risk, and more particularly the symptoms of dependence and the negative consequences associated with alcohol consumption. Therefore, it is crucial not to overlook, in the context of alcohol consumption among students, prevention interventions targeting coping drinking motivations, alcohol-related negative metacognitive beliefs, impulsivity and depressive symptoms. For instance, interventions focusing on coping motives with a framing technique associated with short-term perspective^110,111^, metacognitive beliefs via persuasive communication^112^, impulsivity through cognitive remediation techniques^113^, and depressive symptoms via mindfulness-based interventions^114^ could be effective in mitigating this direct psychological pathway toward AUD in students.

Thus, the findings of this research suggest that, rather than prioritizing interventions targeting either the indirect pathway to AUD or its direct pathway, designing a comprehensive intervention program that combines both sets of interventions would likely be the most effective approach to counter AUD risk among students.”

# **Reviewer 2**

### Suggested Rephrasing and Consistency: Many sections mentions "Prevention of Alcohol Use Disorder (AUD)”. The psychological factors and determinants being studied are risk factors or predictors for AUD. To the reader, the term "prevention" in this context, may imply that interventions can outright prevent AUD. Instead, emphasizing mitigating or modifying the AUD risk would be a preferable alternative just as the conclusion accurately states that identifying these factors enables risk modification or reduction rather than outright prevention.

Thank you for underlining this issue. We share your point of view and have reworded these sentences.

### Introduction:

### Page 5: Similarly, Rephrasing of the last paragraph that "Prevention of Binge Drinking (BD) is tantamount to preventing AUD." This statement oversimplifies the relationship. Instead, emphasize that while reducing BD may mitigate AUD risk, other psychological factors also play a significant role to AUD risk. The above point suggested has been mentioned as concluding statement in the conclusion as well.

We have toned down and reworded these parts.

### Page 5: Inclusion of references to previous studies, especially to discuss the limitations of past research on this topic would be helpful.

Your comment echoes some of those of Reviewer 1. As previously indicated in our responses, we have reworked our introduction to include more clarifications and references to previous studies.

**Main changes made to the manuscript**

- In the *Inter- and intra-individual psychological factors associated with AUD risk* (*Introduction*) section, we further defined the psychological factors studied in this research and gave a brief description of their role in alcohol use disorders (p. 4-5).

“A substantial body of research has identified psychological factors associated with AUD risk in university students, which can be broadly categorized as either inter-individual or intra-individual^12,13^.

Three major inter-individual factors have consistently been linked to AUD risk in young populations. First, *drinking social identity* - the extent to which individuals identify themselves as part of the social category of “drinkers”^14,15^ - positively predicts alcohol use and AUD risk among youth^15–18^. Second, based on Cooper’s motivational model^19^, two external drinking motives, namely *social* (i.e., positive external motives such as drinking to enhance social interactions) and *conformity* (i.e., negative external motives such as drinking to avoid social censure or rejection) *motives* have emerged as strong predictors of alcohol use^16,20^ and AUD risk^19,21–23^. Notably, higher levels of social motives are positively associated with AUD risk, while conformity motives are negatively related to AUD risk. Third, *drinking social norms* - individuals' beliefs about the acceptability and prevalence of alcohol use among peers^24^, influence alcohol use^25^ and AUD risk^26^. Specifically, more positive perceptions of peer drinking are associated with alcohol consumption and increased AUD risk.

Three key intra-individual factors also contribute to AUD risk in young people. First, according to Cooper’s motivational model, two internal drinking motives, namely *enhancement* (i.e., positive internal motive such as drinking to elevate positive mood) and *coping* (i.e., negative internal motives such as drinking to regulate negative affect) *motives* are associated with greater alcohol use and AUD risk in young people^12,16,20,27^. Second, *alcohol-related metacognitions* - schematic information that individuals hold about the significance of their cognitive experience and ways to control it - are significant predictors of AUD risk^28,29^. These metacognitions include positive beliefs about the impact of alcohol use on *cognitive* (helping to control thoughts) and *emotional* (helping to improve mood) *self-regulation*, and negative metacognitions related to the perceived *uncontrollability* and the *negative cognitive functioning impact* of alcohol use. Third, *personality traits and psychopathological variables*, such as loneliness (i.e., a distressing feeling of isolation perception or social rejection^30,31^), anxiety^32^, depression symptoms^33^, and impulsivity (i.e., the tendency to act prematurely without considering consequences) have been consistently associated with alcohol use^34,35^ and AUD risk^31,36,37^. More precisely, impulsivity, as conceptualized in the UPPS model^38^, encompasses dimensions such as *negative* and *positive urgency* (i.e., the tendency to act rashly in intense negative or positive emotional contexts), *lack of perseverance* (i.e., the difficulty to maintain attention on a task), *lack of premeditation* (i.e., the difficulty to plan behaviors and consider their consequences), and *sensation seeking* (i.e., the propensity to look for new and exciting experiences), all of which have been linked to alcohol-related outcomes^39^.

However, it is worth noting that these psychological determinants of AUD risk, and particularly drinking social identity and enhancement motives, are also the strongest predictors of BD^12,26^, suggesting that BD and AUD may share common psychological predictors, that an *indirect psychological pathway* might lead to AUD through BD, and that preventing BD could reduce AUD risk among students.”

- In the section *An indirect psychological pathway from BD to AUD* (*Introduction*), we further justified the place of the psychological factors involved in the indirect pathway to AUD (p. 5-6).

“Social factors are widely recognized as primary initiators of alcohol use in young adults^40–43^, a highly social behavior^44^, characterized by the BD practice, a socially motivated drinking pattern^45^. Indeed, theoretical models, such as Koob and Volkow’s three-stage conceptualization of addiction^46^ and the socioecological framework of drinking contexts^47^, emphasize that excessive alcohol consumption, and particularly BD, is a critical precursor to AUD development. Consistent with these models, research shows that BD is a significant risk factor for AUD^6,7,48^.

BD, as a social drinking pattern, is primarily driven by psychological factors that can be classified into inter-individual (e.g., drinking social identity, drinking norms, social motives) and intra-individual (e.g., positive enhancement motives, sensation seeking) categories. These factors align with the positive affect regulation theory of AUD etiology, which conceptualizes AUD as a maladaptive strategy to enhance positive emotions and social experiences^40^. According to this theory, positive alcohol expectancies - beliefs about alcohol’s ability to improve emotional states and facilitate social bonding - encourage individuals to consume alcohol and increase the likelihood of developing AUD. Supporting this perspective, studies have demonstrated that these BD-related psychological factors, especially interpersonal ones, are indirectly linked to AUD symptoms through higher levels of alcohol consumption^21,49,50^.

Consequently, rather than exerting a direct influence on AUD risk, we hypothesize that these psychological factors primarily promote BD, which in turn increases AUD risk in young adults. In other words, psychological factors associated with the positive affect regulation conceptualization might lead young adults to engage in BD, which subsequently increases their AUD risk. However, existing studies often fail to account for this indirect pathway - and therefore report direct associations - due to two key methodological limitations.”

- In the section *Methodological limitations affecting the understanding of AUD risk* (*Introduction*), we have added elements justifying our interest in the three AUDIT sub-dimensions rather than in the overall AUDIT score (p. 6-8)

“First, research investigating psychological determinants of AUD typically examines these factors in isolation from BD, focusing on their direct effects on AUD risk without considering the mediating role of BD. This approach overlooks the possibility that BD may serve as a mediator between some psychological factors (e.g., drinking identity, social motives) and AUD risk^21,50^, potentially explaining why many determinants appear common to both BD and AUD risk.

Second, most studies predicting AUD risk among university students use the overall AUDIT score as the outcome variable. According to the original authors, AUDIT measures three major dimensions associated with AUD risk: alcohol intake, alcohol dependence symptoms, and alcohol-related problems^51^. However, these three sub-dimensions (i) correspond to different aspects of alcohol consumption^9,10,51,52^, (ii) have different predictors and causal factors^53^, and (iii) are themselves different predictors of drinking behavior and its consequences^54^. Notably, the “alcohol intake” dimension is highly correlated with BD, as evidenced by the sensitivity and specificity of AUDIT-C in detecting BD^55,56^. In contrast, the other two dimensions - dependence symptoms and alcohol-related problems - capture distinct aspects of AUD risk from excessive consumption^9,10^. Consequently, studies relying on the overall AUDIT score may overestimate the role of BD-related psychological determinants in AUD risk and underestimate factors more closely tied to dependence symptoms and alcohol-related problems. Therefore, a growing body of evidence suggests that the AUDIT is not a unitary scale but should rather be distinguished according to its sub-dimensions to investigate the risk of AUD^9–11,57,58^.

Thus, these two methodological issues may lead to (i) an overestimation of AUD predictors that are shared with BD but primarily act indirectly through BD, and (ii) an underestimation of predictors less correlated with BD but directly influencing AUD risk. Indeed, when focusing specifically on dependence symptoms and alcohol-related problems, some evidence suggests that specific psychological factors are associated with AUD risk among youth, highlighting a direct psychological pathway to AUD, independent from BD.”

- In the section *A direct psychological pathway to AUD* *risk* (*Introduction*), we further justified the role of the psychological factors involved in the direct pathway to AUD (p. 8-9)

“One of the most prominent theories of AUD etiology suggests that AUD develops as a way to alleviate negative affect, such as reducing anxiety, stress, and depression^27,59–62^. Many young adults report drinking to cope with negative emotions^26,63,64^. Psychological factors associated with this negative affect regulation process - such as negative reinforcement motives (i.e., coping motives), negative alcohol-related metacognitions, and specific personality traits - may play a direct role in AUD risk, independent of BD, which would be less influenced by these factors.

Empirical evidence supports this perspective. Studies focusing on the prediction of dependence symptoms and alcohol-related problems - rather than overall AUDIT scores - reveal notable differences between psychological predictors of BD and those of AUD risk.

Regarding drinking motives, while BD is primarily explained by social and enhancement motives, coping motives are more strongly associated with alcohol-related problems^27,65^ and dependence symptoms^32,66^. Thus, coping motives appear to exert a direct influence on AUD risk, independent of BD practice.

Regarding alcohol-related metacognitions, empirical research highlights distinct roles for positive and negative metacognitions. Positive metacognitions, such as emotional self-regulation, are primarily associated with BD^28^. Conversely, negative metacognitions (e.g., beliefs about uncontrollability and negative consequences of alcohol use) are crucial for the persistence of alcohol consumption^67,68^ and are significant predictors of alcohol dependence ^69^. These findings suggest that negative metacognitions play a direct role in AUD risk among university students, independent of BD practice.

Regarding personality traits, the predictive role of impulsivity dimensions differs between alcohol use and alcohol-related problems or dependence symptoms. While the lack of perseverance is more strongly associated with alcohol use, negative urgency and the lack of premeditation are more predictive of AUD risk^39^. Additionally, loneliness is a characteristic specific to higher AUD risk compared to lower dependence risk^31^, and depression symptoms have been more strongly associated with alcohol dependence symptoms than with general alcohol use^33^. These findings indicate that some impulsivity dimensions (e.g., negative urgency and lack of premeditation), as well as loneliness and depression, may directly contribute to AUD risk among university students, beyond their association with BD.”

### Materials and Methods

### -Procedures and Participants:

### Page 8: Eligibility/Selection Criteria: Authors may expand on the eligibility and inclusion criteria of the sample selected for the study. This will help readers better interpret the study's findings and understand its applicability.

The only inclusion criterion for taking part in the study was to be a Caen Normandie University student of legal age (18). To be included in the final sample, participants were required to have consumed alcohol in the last 12 months and to have completed all the variables of interest in the study. We have indicated these elements in the revised manuscript.

**Main changes made to the manuscript**

- In the *Procedure and participants* (*Method*) section, we have indicated that the only inclusion criterion for taking part in the study was to be a Caen Normandie University student of legal age (18). (p. 11)
- In the *Procedure and participants* (*Method*) section, we have added a figure (Fig. 2) representing the selection process for our final sample. (p. 11)

**Fig 2. Flow diagram**

### Statistical Analysis:

### Page 14: Typological and Grammatical error. Repetition of sentences.

We have corrected this in the revised manuscript.

### Result: The tables provided are comprehensive and effectively summarize the key findings. However, a mediational diagram or model illustrating the relationships between psychological predictors, binge drinking as a mediator, and AUD outcomes could aid in understanding the mediation pathways. It may be included to enhance the presentation of the results.

As indicated in our response to the editor, we have added a figure (Fig. 3) to our revised manuscript to improve the presentation of our results.

**Main changes made to the manuscript**

- In the *Results* section, we have added a figure (Fig. 3) showing the main results of our statistical model (p. 18).

### Limitations and Future Directions:

### - Page 20: Typological error- “Econd” instead of Second.

We have corrected the error.

### - Socio-Demographic Insights: Acknowledge the socio-demographic imbalance, particularly the higher percentage of female respondents (67.2%) and discuss potential implications of this gender distribution on the findings.

Thank you for your comment, which echoes one of those made by the Editor. In addition to our response made to the Editor’s comment, and while we know that there are differences in alcohol consumption between men and women, we believe that this gender distribution did not have a significant impact on our results because we were careful to control for the effect of gender in our statistical model (p. 16).
